# MicroRNA-29c-3p and -126a Contribute to the Decreased Angiogenic Potential of Aging Endothelial Progenitor Cells

**DOI:** 10.3390/ijms26094259

**Published:** 2025-04-30

**Authors:** Derek M. Dykxhoorn, Andrea Da Fonseca Ferreira, Karenn Gomez, Jianjun Shi, Shoukang Zhu, Lukun Zhang, Huilan Wang, Jianqin Wei, Qianhuan Zhang, Conrad J. Macon, Joshua M. Hare, George R. Marzouka, Liyong Wang, Chunming Dong

**Affiliations:** 1 John T. Macdonald Foundation Department of Human Genetics and, the John P. Hussman Institute for Human Genomics, University of Miami Miller School of Medicine, Miami, FL 33136, USA; ddykxhoorn@med.miami.edu (D.M.D.);; 2 Interdisciplinary Stem Cell Institute, University of Miami Miller School of Medicine, Miami, FL 33136, USA; axd1272@med.miami.edu (A.D.F.F.);; 3 Department of Medicine, University of Miami Miller School of Medicine, Miami, FL 33136, USA

**Keywords:** aging, angiogenesis, endothelial progenitor cells

## Abstract

EPCs play important roles in the maintenance of vascular repair and health. Aging is associated with both reduced numbers and functional impairment of EPCs, leading to diminished angiogenic capacity, impaired cardiac repair, and increased risk for cardiovascular disease (CVD). The molecular mechanisms that govern EPC function in cardiovascular health are not fully understood, but there is increasing evidence that microRNAs (miRNAs) play key roles in modulating EPC functionality, endothelial homeostasis, and vascular repair. We aimed to determine how aging alters endothelial progenitor (EPC) health and functionality by altering key miRNA-mRNA pathways. To identify key miRNA-mRNA pathways contributing to diminished EPC functionality associated with aging, microRNA and mRNA profiling were conducted in EPCs from young and aged C57BL/6 mice. We identified a complex aging-associated regulatory network involving two miRNAs—miR-29c-3p and -126a—that acted in tandem to impair vascular endothelial growth factor signaling through targeting Klf2 and Spred1, respectively. The modulation of components of the miR-29c-3p–Klf2–miR-126a–Spred-1–Vegf signaling pathway altered EPC self-renewal capacity, vascular tube formation, and migration in vitro, as well as cardiac repair in vivo. The miR-29c-3p–Klf2–miR-126a–Spred1–Vegf signaling axis plays a critical role in regulating the aging-associated deficits in EPC-mediated vascular repair and CVD risk.

## 1. Introduction

Aging is a significant risk factor for vascular disease. Aging-associated vascular cell senescence contributes to the pathogenesis of atherosclerosis by disrupting the balance between vascular injury and repair [1]. Vascular repair involves local repair with proliferation and in-migration of mature endothelial cells (ECs) or the recruitment of Endothelial Progenitor Cells (EPCs), which have regenerative capability and the ability to differentiate into mature ECs, to the sites of injury [2]. Although there are no settled molecular signatures to define circulating EPCs, it is agreed that EPCs are enriched in lineage negative bone marrow cells (lin^−^ BMCs) [3]. We use endothelial progenitor cells (EPCs) to refer to the population of lin^−^ BMCs capable of de novo vessel formation [4]. In response to vascular damage or inflammation, EPCs migrate from the bone marrow to the site of vascular injury to halt the inflammatory process and preventing atherosclerosis [5,6,7,8,9]. Circulating EPCs contribute to vascular repair by halting the inflammatory process and by activating local ECs through the secretion of proangiogenic growth factors [5]. EPCs exhibit substantially decreased self-renewal, migratory, and differentiation potential during aging [6].

MicroRNAs (miRNAs) play important roles in progenitor cell function and health [7]. Recently, we showed that dysregulation of miR-21/miR-10A*-Hmga2 (High-mobility group AT-hook 2) and miR-146a—Plk2 (Polo-like Kinase 2) contributes to EPC senescence and impaired angiogenesis in vivo [4,5,6,7,8]. Given the multiplicity of phenotypes associated with vascular injury and repair, we sought to identify additional miRNAs that contribute to impaired angiogenesis due to EPC aging. MiR-29c-3p and miR-126a both showed differential expressions between young and aged mouse EPCs. MiR-29c-3p targets *Klf2* (Kruppel-like Factor 2) previously shown to be induced in response to hemodynamic forces in ECs promoting angiogenesis [9]. Further, we showed that Klf2 upregulates the EC-specific miR-126a expression which, in turn, regulated Spred-1 expression to modulate Vegf signaling consistent with a previous study [10]. Our study demonstrated the important role of the ↑miR-29c-3p → ↓Klf2 → ↓miR-126a → ↑Spred1 → ↓VEGF signaling pathway in maintaining EPC functionality in angiogenesis and cardiac repair during aging.

## 2. Results

### 2.1. MiR-29c-3p-Klf2 Is a miRNA-mRNA Pair Dysregulated During EPC Aging

**Figure 1:**
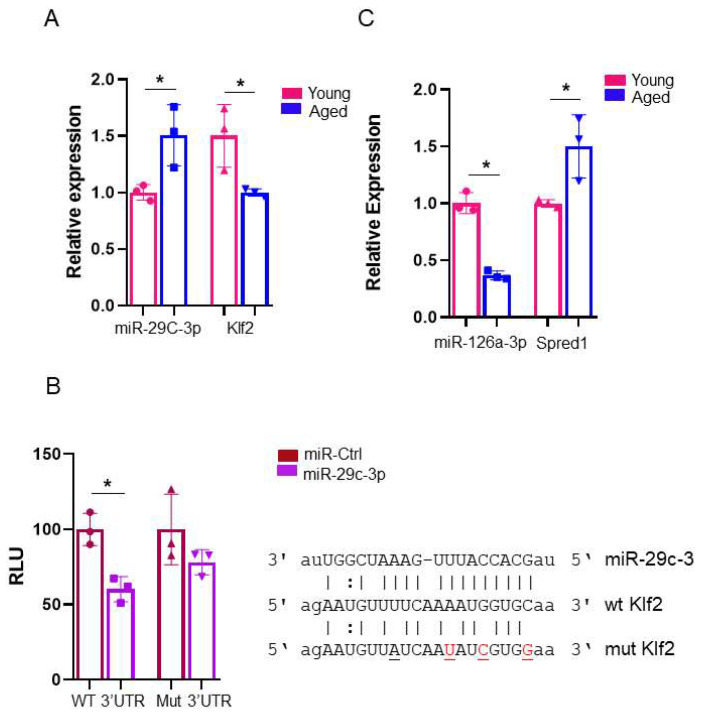
MiR-29c-3p-Klf2 and miR-126a-Spred1 are miRNA-mRNA pairs dysregulated during lin-BMCs aging. (**A**) MiR-29c-3p expression was elevated in EPCs derived from aged compared to young mice, while its putative target Klf2 shows the inverse pattern of expression as measured by qRT-PCR (mean ± SD; * *p* < 0.05). Graph shows three biological replicates, i.e., average assessments for three different cell wells. Test used: *t*-test. (**B**) Mir-29c-3p targeted Klf2 through the predicted miRNA binding site in its 3′ UTR (WT 3′UTR). Reporter gene constructs with WT 3′UTR or Mut 3′UTR bearing mutations in the miR-29c binding site were co-transfected with miR-29c-3p or a scrambled miRNA (miR-Ctr). Luciferase activities were measured 48 h after transfection and the values represent the Gaussia luciferase/secreted alkaline phosphatase ratios (RLU) (mean ± SD; * *p* < 0.01). Graph shows three biological replicates, i.e., average assessments for three different cell wells. Test used: *t*-test. (**C**) Mir-126a expression was decreased in EPCs derived from aged compared to young mice while its target gene Spred-1 was elevated in aged compared to young EPCs as determined by qRT-PCR (mean ± SD; * *p* < 0.05). Graph shows three biological replicates, i.e., average assessments for three different cell wells. Test used in all graphs: *t*-test. EPCs isolated from male C57BL/6 mice bone marrow were used in these assays.

Previously, we identified 38 miRNAs and 1135 mRNAs that were differentially expressed between young and aged EPCs [4,5,6,7,8]. We found that miR-29c-3p was increased while *Klf2* was decreased in aged EPCs [8], which was validated by qRT-PCR (Figure 1A). Furthermore, *Klf2* is a predicted target of miR-29c-3p by in silico analysis, which was confirmed by the 3′UTR reporter gene analysis (Figure 1B).

### 2.2. Establishing the miR-29c-3p–Klf2–miR-126a–Spred1-Vegf Axis

In studies of blood flow induced angiogenesis [11] and endotoxin-induced vascular injury [12], it was shown that Klf2 upregulates the expression of an endothelial-specific miRNA, miR-126a which regulates EC function and angiogenesis by inhibiting Spred-1 (Sprouty-related Ena/VASP homology-1 domain-containing protein) expression. Spred-1 negatively regulates growth factor- and cytokine-induced ERK (Extracellular signal-regulated kinase) activation and acts as an endogenous inhibitor of Vegf signaling [13,14]. Therefore, miR-126a and Spred-1 could be the downstream targets of miR-29c-3p and contribute to EPC aging by suppressing Vegf signaling. Indeed, miR-126a was decreased and Spred-1 was upregulated in aged EPCs (Figure 1C). Therefore, we hypothesize that during EPC aging, miR-29c-3p silences *Klf2* expression, which reduces miR-126a expression relieving the repression on *Spred-1* expression which, in turn, suppresses Vegf signaling.

**Figure 2:**
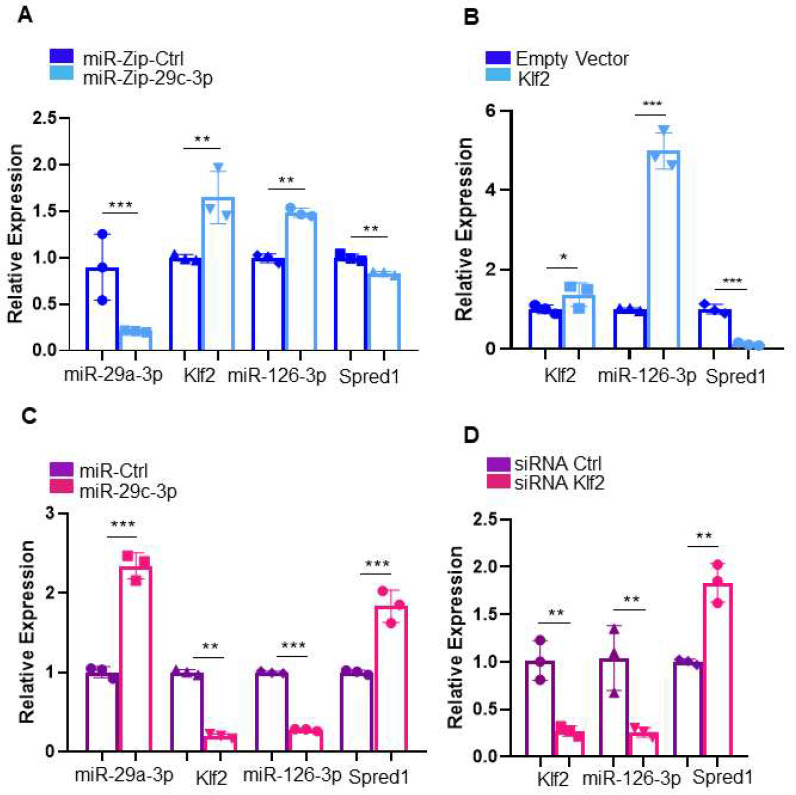
miR-29c-3p and miR-126a regulate Klf2 and Spred-1 in tandem in EPCs. (**A**) The expression levels of miR-29c-3p, Klf2, miR-126a, and Spred1 were measured by qRT-PCR in aged EPCs 48h following transduction of lentivirus vector containing the miRNA antagonist of miR-29c-3p (miRZip-29c) or a scrambled control antagomiR (miRZip-Ctr). MirZip-29c leads to a reduction in miR-29c-3p expression with a concomitant increase in Klf2 and its downstream transcriptional target miR-126a that, in turn, decreased Spred1 expression (mean ± SD; ** *p* < 0.01; *** *p* < 0.001). Graph shows three biological replicates, i.e., assessments for three different cell wells. Test used: *t*-test. (**B**) Over-expression of Klf2 lacking the 3′ UTR in aged EPCs resulted in increased miR-126a and decreased Spred-1 expression compared to empty vector control (mean ± SD; * *p* < 0.05; *** *p* < 0.001). Graph shows three biological replicates, i.e., average assessments for three different cell wells. Test used: *t*-test. (**C**) Overexpression of miR-29c-3p in young EPCs led to decreased Klf2 and miR-126a and increased Spred1 compared to control miRNA (miR-Ctr) (mean ± SD; ** *p* < 0.01; *** *p* < 0.001). Graph shows three biological replicates, i.e., assessments for three different cell wells. Test used: *t*-test. (**D**) Silencing of Klf2 via siRNA in young EPCs led to decreased miR-126a and increased in Spred1 (** *p* < 0.01). Graph shows three biological replicates, i.e., average assessments for three different cell wells. Test used: *t*-test. EPCs isolated from male C57BL/6 mice bone marrow were used in these assays.

**Figure 3:**
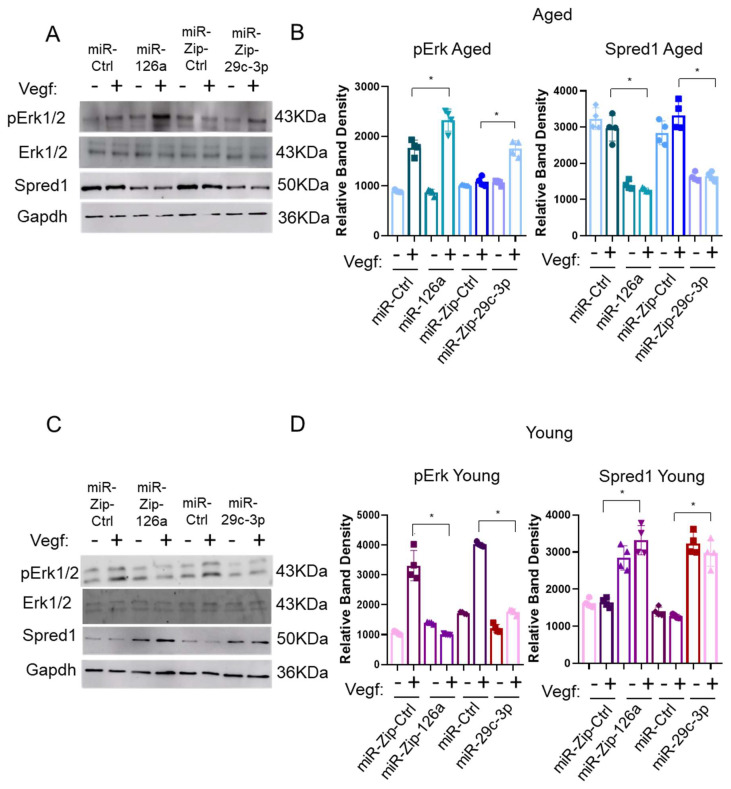
miR-29c-3p and miR-126a regulates VEGF signaling in EPCs by repressing Klf2 and Spred1, respectively. (**A**,**B**) Representative images of Immunoblots from aged EPCs modified with miR-Ctrl, miR-126a, miR-Zip-Ctrl, miR-Zip-29c-3p. Phosphorylation of ERK was induced by VEGF in aged EPCs transduced with miR-126a and miR-Zip-29c. Conversely, Spred 1 levels were reduced in miR-126a and miR-Zip-29c in the same condition. (**C**,**D**) Immunoblot of lysates from young EPCs infected with miRZip control (miRZip-Ctr), miRZip-126a, miRNA control (miR-Ctr), or miR-29c-3p in the presence or absence of VEGF (10 ng/mL, 10 min). VEGF induced phosphorylation of ERK (pErk1/2) was blocked by miR-126a knockdown and miR-29c-3p overexpression. In addition, both the overexpression of miR-29c-3p and silencing of miR-126a enhanced Spred1 expression but not effect on ERK1/2 protein levels. (**B**) Quantification of pERk1/2 and Spred-1 protine in (**A**). GAPDH was used as loading control for both aged and young animal groups. EPCs isolated from male C57BL/6 mice bone marrow were used in these assays (* *p* < 0.05). Graph shows four biological replicates, i.e., assessments for four different cell wells. Test used: *t*-test. EPCs isolated from male C57BL/6 mice bone marrow were used in these assays.

To determine if miR-126a and *Spred-1* expression was governed by miR-29c-3p, through *Klf2*, we performed a series of knockdown and overexpression studies in young and aged EPCs. Knocking down miR-29c-3p expression in aged EPCs led to increased *Klf2* mRNA and miR-126a expression and decreased *Spred-1* expression at the mRNA (Figure 2A) and protein (Figure 3A,B) levels. Conversely, overexpression of miR-29c-3p in young EPCs led to a decrease in *Klf2* mRNA and miR-126a expression and increased *Spred1* expression [mRNA (Figure 2C) and protein (Figure 3C,D) levels]. Similarly, overexpressing *Klf2* in aged EPCs led to higher miR-126a and decreased *Spred-1* mRNA levels (Figure 2B). Conversely, knocking down *Klf2* in young EPCs led to decreased miR-126a and increased *Spred-1* mRNA expression (Figure 2D).

To establish the central role of the ↑miR-29c-3p → ↓Klf2 → ↓miR-126a → ↑Spred1 → ↓VEGF signaling pathway during EPC aging, we assessed the activity of the Vegf signaling pathway by monitoring p-ERK1/2 levels in Vegf treated EPCs. Overexpression of miR-126a or decreased miR-29c-3p (miRZip-29c-3p) in aged EPCs led to reduced expression of Spred-1 and increased Vegf responsiveness (increased p-Erk1/2 levels) (Figure 3A,B). Silencing miR-126a (miRZip-126a) or elevated miR-29c-3p expression in young EPCs resulting in decreased p-Erk 1/2 levels (Figure 3C,D). Therefore, the modulation of the miR-29c-3p → Klf2 → miR-126a → Spred-1 axis modulates Vegf signaling.

### 2.3. The miR-29c-3p → ↓Klf2 → ↓miR-126a → ↑Spred1 → ↓VEGF Signaling Pathway Regulates the Self-Renewal Potential, Vascular Tube Formation, and Migration of EPCs

**Figure 4:**
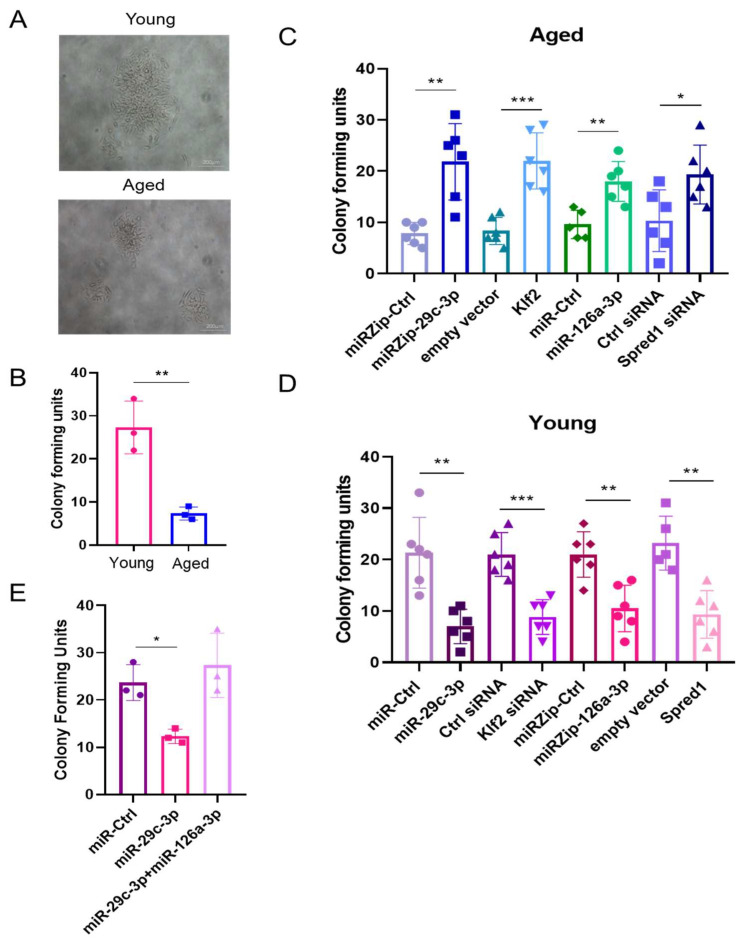
miR-29c-3p and miR-126a overexpression or knockdown regulates EPC self-renewal through Klf2 and Spred-1. EPCs from 3-week-old male C57BL/6 mice bone marrow (young) mice had significantly higher colony formation compared to 18-month-old (aged) male C57BL/6 mice (scale bars = 20 μm). (mean ± SD; ** *p* < 0.01). Graph shows three biological replicates, i.e., assessments for three different cell wells. Test used: *t*-test (**A**,**B**). (**C**) The silencing of miR-29c-3p, overexpression of Klf2, overexpression of miR-126a, or the silencing of Spred-1 dramatically improved aged EPCs colony formation compared to their respective control treatments. Graph shows six biological replicates, i.e., assessments for six different cell dishes (**D**) Conversely, the overexpression of miR-29c-3p, siRNA mediated silencing of Klf2, miRZip-126a silencing, and Spred-1 overexpression reduced the colony formation potential of young EPCs (mean ± SD; ** *p* < 0.01;*** *p* < 0.001). The graph shows six biological replicates, i.e., assessments for six different cell dishes. Test used: *t*-test. (**E**) miR-126a overexpression abolished the inhibitory effect of miR-29c-3p on the self-renewal potential of young EPCs suggest that miR-126a acts downstream of miR-29c-3p (mean ± SD; * *p* < 0.05). The graph shows three biological replicates, i.e., assessments for three different cell dishes. Test used: ANOVA followed by Dunnett’s multiple comparisons test. EPCs isolated from male C57BL/6 mice bone marrow were used in these assays. Scale bars: 200 µm.

**Figure 5:**
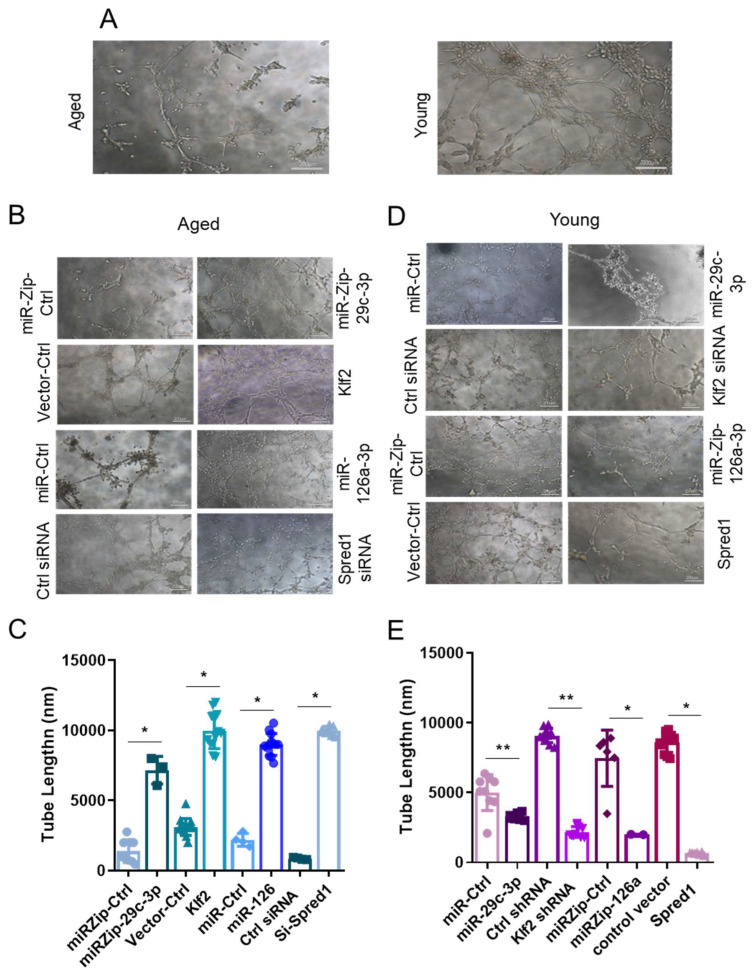
miR-29c-3p and miR-126a overexpression or knockdown regulates EPC angiogenesis through Klf2 and Spred-1 in vitro. (**A**) Vascular tube formation in young and aged EPCs. (**B**,**C**) Aged EPCs transduced with lentivirus expressing a miR-29c-3p antagonist (miRZip-29c-3p), Klf2, miR-126a, or Spred-1 silencing (Spred-1 shRNA) showed significantly improved vascular tube formation, as measured by vascular tube length, compared to their respective controls (mean ± SD; * *p* < 0.05). The graph shows six biological replicates, i.e., assessments for six different cell dishes. Test used: *t*-test. (**D**,**E**) Young EPCs transduced with lentivirus encoding miR-29c-3p, silencing of Klf2 (Klf2 shRNA), a miR-126a agonist (miRZip-126a), or overexpression of Spred1 led to reduced capillary tube formation capacity on matrigel compared to the appropriate control treated samples (mean ± SD; * *p* < 0.05; ** *p* < 0.01). Graphs (**C**,**E**) show three biological replicates, i.e., assessments for three different cell dishes with 3 to 12 random fields evaluated. Test used: *t*-test. EPCs isolated from male C57BL/6 mice bone marrow were used in these assays. Scale bars: 200 µm.

**Figure 6:**
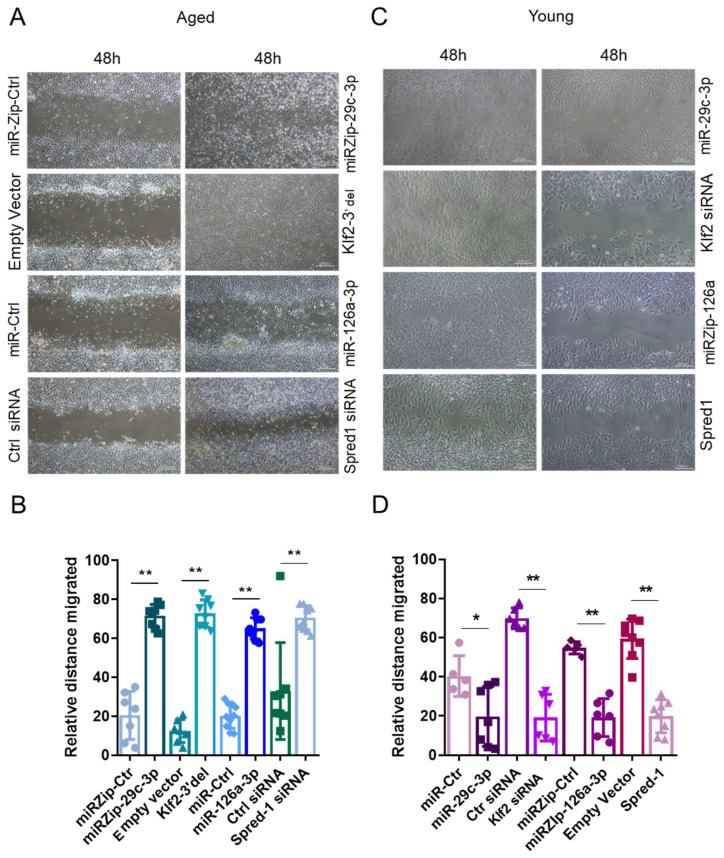
miR-29c-3p and miR-126a overexpression or knockdown regulates EPCs migration through Klf2 and Spred1 in vitro. (**A**,**B**) Representative images and quantitation of wound healing in aged EPCs after 48h postscratch. The silencing of miR-29c-3p (miRZip-29c), overexpression of miR-126a, Klf2 overexpression, or the siRNA-mediated silencing of Spred1 enhanced wound healing capacity of EPCs from aged mice compared to their respective controls. (mean ± SD; ** *p* < 0.01). The graph shows three biological replicates, i.e., assessments of three different cell wells with seven to eight random fields. Test used: *t*-test. (**C**,**D**) Representative images of the wound healing assay in young EPCs at 48h post scratch. The overexpression of miR-29c-3p, the inhibition of miR-126a, the siRNA-mediated silencing of Klf2, or the overexpression of Spred-1 impaired the wound healing ability of young EPCs compared to their respective control treatment (mean ± SD; * *p* < 0.05; ** *p* < 0.01). The graph shows three biological replicates, i.e., assessments of three different cell wells with seven to eight fields. Test used: *t*-test. EPCs isolated from male C57BL/6 mice bone marrow were used in these assays. Scale bars: 200 µm.

We assessed the impact of modulating components of the ↑miR-29c-3p → ↓Klf2 → ↓miR-126a → ↑Spred1 → ↓VEGF pathway on colony formation (self-renewal), tube formation (vasculogenesis), and wound healing (migration). Compared to young EPCs, aged EPCs had decreased CFUs (Figure 4A,B) and impaired capability to form vascular tubes (Figure 5A). In aged EPCs, the silencing of miR-29c-3p (miRZip-29c-3p), overexpression of Klf2 or miR-126a, and Spred-1 knockdown (Spred-1 siRNA) led to a significant increase in CFUs (Figure 4C), improved capillary tube formation, as measured by vascular tube length (Figure 5B,C) and number (Supplemental Figure S1A), as well as increased migratory capability (Figure 6A,B). Conversely, the overexpression of miR-29c-3p, downregulation of Klf2 (Klf2 siRNA) or miR-126a (miR-Zip-126a), and the overexpression of Spred-1 in young EPCs led to a significant decrease in CFUs (Figure 4D), impairment capillary tube formation (Figure 5D,E, Supplemental Figure S1B), and reduced migratory capacity (Figure 6C,D). Interestingly, the co-expression of miR-126a with miR-29c-3p blocked the miR-29c-3p mediated reduction in CFUs in young EPC (Figure 4E), providing additional evidence that miR-126a works downstream of miR-29c-3p. These results suggest that the ↑miR-29c-3p → ↓Klf2 → ↓miR-126a → ↑Spred1 → ↓VEGF is important for regulating multiple functional aspects of EPCs.

### 2.4. Modulation of the ↑miR-29c-3p → ↓Klf2 → ↓miR-126a → ↑Spred1 → ↓VEGF Signaling Pathway Impacts Cardiac Repair In Vivo

**Figure 7:**
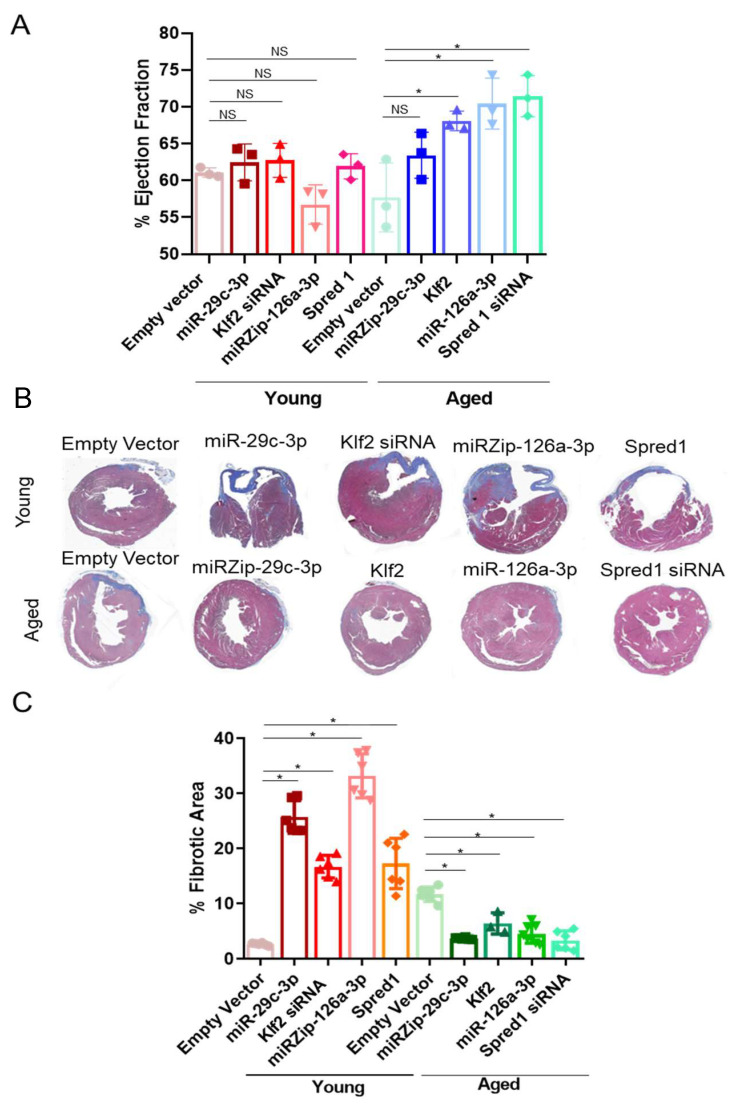
miR-29c-3p and miR-126a regulate cardiac repair in vivo. Mice with acute MI were treated with EPCs with different modifications. LVEF (**A**) and Fibrosis (**B**,**C**) were evaluated for 4 weeks post-MI. (**A**). Young EPCs with overexpression of miR-29c-3p, silencing of Klf2 (Klf2 shRNA), silencing of miR-26a (miRZip-126a), or Spred1 overexpression resulted in a decrease in the left ventricle ejection fraction (EF) compared to empty vector. Conversely, aged EPCs with silencing of miR-29c-3p (miRZip-29c), Klf2 expression, miR-126a expression, or siRNA-mediated Spred1 silencing enhanced the EF compared to empty vector (mean ± SD; * *p* < 0.05). The graph shows three biological replicates, i.e., assessments for three different animals. Test used: ANOVA followed by Dunnett’s multiple comparisons test. (**B**) Representative images of heart cross-section of each treatment group are shown. (**C**) Fibrotic area was quantified. Young EPCs overexpressing miR-29c-3p, silencing of Klf2 (Klf2 shRNA), miR-126a silencing (miRZip-126a), or Spred-1 overexpression resulted in increased fibrosis compared to the empty vector. Aged EPCs with lentiviral vectors that silenced miR-29c-3p (miRZip-29c), elevated Klf2 expression, miR-126a overexpression, or Spred-1 silencing (Spred-1 shRNA) led to a significant decrease in fibrosis compared to the empty vector (mean ± SD; * *p* < 0.05). Graph shows three biological replicates, i.e., assessments for three different animals with three to six fields. Test used: ANOVA followed by Dunnett’s multiple comparisons test.

To examine the role of miR-29c-3p → ↓Klf2 → ↓miR-126a → ↑Spred1 → ↓VEGF in EPC-mediated cardiac repair in vivo, a mouse model of acute myocardial infarction (AMI) with LAD ligation was used. Successful coronary artery occlusion was verified by ST segment elevation on electrocardiogram (ECG). AMI mice received EPCs with different modifications and echocardiography was performed at baseline, 24 h, and 4 weeks post-LAD ligation. There was no significant difference between any of the groups at baseline or at 24 h post-LAD ligation. Significant improvement in Left ventricular ejection fraction (LVEF) were detected 4 weeks post ligation in mice receiving aged EPCs transduced with Klf2, miR-126a, and Spred1 siRNA compared with mice treated with aged cells transduced with the empty vector (Figure 7A) while no statistically significant difference was detected among mice receiving young EPCs with different modifications compared to young EPCs transduced with the empty vector (Figure 7A).

At 4 weeks post LAD ligation, infarction, angiogenesis, and apoptosis were also evaluated. Mice receiving aged cells transfected with miRZip-29c, Klf2, miR-126a, or Spred-1 siRNA had—significantly less fibrosis (Figure 7B,C), increased microvascular density (Supplemental Figure S2A), and reduced apoptosis (Supplemental Figure S3A) compared to aged cells transduced with empty vectors. On the other hand, mice receiving young EPCs transduced with miR-29c, Klf2 siRNA, miRZip-126a, or Spred-1 have greater fibrosis (Figure 7B,C), no significant change in angiogenesis (Supplemental Figure S2B), and increased apoptosis (Supplemental Figure S3B), compared with empty vector transduced young EPCs. These experiments suggest that genetically modification of the miR-29c-3p → Klf2 →miR-126a → Spred1 axis rejuvenate aged EPCs promoting cardiac repair following AMI.

## 3. Discussion

A key driver of age-associated CVD risk is the impairment of vascular repair [15]. Progenitor cells can divide and differentiate into specific cell types to replenish and repair damaged tissues. However, this regenerative capacity of progenitor cells decreases with age in many tissues [16,17,18]. For example, there is a significant decrease in EPC quantity and functionality with aging, contributing to compromised vascular integrity and CVD [19,20,21]. Identifying the underlying mechanisms for EPC senescence can guide the development of effective therapeutic approaches to reduce age-associated CVD risk.

MiRNAs play key roles in the regulation of a wide variety of biological processes [22]. In this study, we identified a novel pathway with two miRNAs—miR-29c-3p and miR-126a—working in tandem to regulate Vegf signaling. MiR-29c-3p was shown to be upregulated while miR-126a was down regulated in aged compared to younger EPCs. The upregulation of miR-29c-3p was shown to directly silence the expression of the transcription factor Klf2 that has been shown to be a key regulator of miR-126a expression. Further, this miR-126a silencing relieved the repression of Spred-1, a negative regulator of Vegf signaling, a critical determinant of angiogenic capacity crucial for proper tissue repair in response to injury, ischemia, and wound healing [23]. We show the aging-associated upregulation miR-29c-3p drives a cascade of events that leads to impaired Vegf signaling and decreased EPC functionality in vitro and in vivo according to the following pathway: ↑miR-29c-3p → ↓Klf2 → ↓miR-126a → ↑Spred1 → ↓VEGF signaling pathway. Down-regulation of miR-29c and Spred-1 or up-regulation of miR-126a and Klf 2 led to improved EPC self-renewal, vascular tube formation, and migration, as well as promoting in vivo cardiac repair (AMI mouse model). Reciprocally, the overexpression of miR-29c-3p and Spred-1 or the silencing of miR-126a and Klf2 in young EPCs impaired their functionality in vitro and in vivo.

Our findings are consistent with miR-126a playing a key role in EC-mediated vascular repair and angiogenesis. MiR-126a plays a critical role in endothelial cell proliferation, migration, and angiogenesis, as well as response to injury [14]. Targeted deletion of miR-126a in mice impairs vascular integrity and reduced angiogenesis following MI [14]. Within developing aortic arch blood vessels of zebrafish, Klf2 upregulated miR-126a expression leading to VEGF-induced angiogenesis through the suppression of Spred-1 expression [11]. Further, miR-126a protects against vascular injury by promoting EPC migration/homing of EPCs to sites of injury in rats [24]. Interestingly, Chu et al. (2017) showed that miR-126a silencing stimulated lipid synthesis in mammary luminal epithelial cells (MECs) by increasing the levels of several lipid synthesis enzymes—FASN, ACSL1, and Insig1 [25]. These findings open the possibility that miR-126a-mediated alterations in lipid metabolism-associated genes may work in concert with the modulation of VEGF signaling to drive CVD susceptibility. Our study not only provided additional evidence supporting the protective role of miRNA-126a in vascular repair but also identified important upstream regulators of miRNA-126a in EPCs.

Several studies suggest that miR-29c plays a multifactorial role in cardiac health. For example, miR-29c directly targeted insulin growth factor 1 (IGF-1) in HUVECs resulting in reduced migration and tube formation [26]. MiR-29c may be a potential biomarker for cardiovascular diseases, including coronary heart disease, heart failure, and chronic obstructive pulmonary disease (COPD) [27,28]. MiR-29c was responsive to hypoxia-inducible factor (HIF)-1. Upregulated miR-29c suppressed Serca2 expression and the uptake of Ca2+ to reduce cardiomyocyte contractility [29]. MiR-29c was also shown to be upregulated in response to TGFβ and target the histone lysine methyl transferase 5 (KMT5) altering H4K20me3 levels and inducing cardiac aging [30]. Our study is the first to show that miR-29c expression modulates angiogenesis by regulating the expression of a second miRNA, miR-126a, through repressing the transcriptional activator Klf2. Based on the centrality of miR-29c-3p and miR-126a on cardiovascular health, the miR-29c-3p → ↓Klf2 →↓miR-126a → ↑Spred1 →↓VEGF signaling pathway may serve as a potent target pathway for the regulation of EPC functionality and cardiac repair.

MiRNAs are key regulators of gene expression, and their dysregulation has been shown to be involved in various pathological states, including cardiovascular disease. As such, the modulation of miRNA expression levels has been promoted as a potential therapeutic approach for a variety of diseases [31]. This is especially the case since an individual miRNA can modulate multiple mRNA targets and thus could potentially mitigate the effects of multiple pathways contributing to the pathogenesis of disease [32]. These approaches could include the delivery of miRNA mimics, miRNA inhibitors, or miRNA sponges. Recent studies point to the potential of modulating miRNA-mRNA pathways for therapeutic outcomes [32]. However, several challenges still need to be overcome for the effective implementation of these approaches into the clinic. These include issues surrounding the cell type specific delivery of miRNA modulatory molecules and the potential for off target toxicities [33,34]. Although these are challenges to the implementation of miRNA-based targeted therapies, as the field of miRNA biology matures, so will the approaches for their effective implementation as therapeutic strategies. An increased understanding of the specific role(s) of miRNA-mRNA pathways in cell biology will not only identify their role in disease pathology but also unlock potential off target effects of these molecules. By understanding the breadth of targeting of these molecules, it may also be possible to develop approaches to mitigate the unintended off target effects increasing their therapeutic utility [15].

## 4. Methods

### 4.1. Animals

Male C57BL/6 mice were used for this study. Wild-type (wt) 6–8 week (wks) old mice were purchased from Jackson Laboratory (Strain # 000664) (Bar Harbor, ME, USA). Aged wt mice (>29 months) were purchased from the National Institute on Aging (Bethesda, MD, USA). Mice were euthanized at 6 months and >2.5 years, respectively, and total bone marrow cells were collected. Euthanasia was performed according to approved guidelines: 5 animals at a time were placed inside the CO2 chamber that had a fill rate between 30 and 70% of the chamber per minute. According to the measurements of our chamber and appropriate automatic sets, CO_2_ flow was stopped after 6 min and animals showing no sign of breathing were left in the chamber for 10 more minutes. Cervical dislocation was used as a secondary method of euthanasia.

### 4.2. Quantitative RT-PCR

RNA was isolated using the miRNeasy Mini kit (Qiagen, Germantown, MD, USA). MiRNAs and mRNAs were quantified using TaqMan^®^ MicroRNA Assay kits and Taqman^®^ Gene Expression Assays, respectively (Thermo Fisher Scientific, Waltham, MA, USA). miRNA expression was normalized to the U6 small nucleolar RNA while mRNAs were normalized to the 18S ribosome gene (Thermo Fisher Scientific, Waltham, MA, USA). Each qRT-PCR reaction was performed in duplicate, and analysis was performed using the 2^−ΔΔCT^ method.

### 4.3. MiRNA and mRNA Profiling Analysis

Differential microRNA and mRNA expression in young vs. aged mice EPCs was previously described [8]. MiRNA target genes were identified using miRNA databases, including TargetScan and miRDB [35,36]. Pearson correlations and *p*-values were calculated for all miRNA–mRNA pairs with Benjamini–Hochberg false discovery correction used to adjust for multiple comparisons [37].

### 4.4. Validating Potential miRNA-mRNA Regulatory Relationships in 293T Cell Lines

Regulatory effects of miR-29c-3p on Klf2 expression were validated by luciferase reporter assay in HEK293T cells using the Klf2 3′UTR (with or without mutations in the predicted miR-29c-3p binding sites) cloned downstream of the Gaussian luciferase gene in the pEZX-GA02 vector (GeneCopoeia, Rockville, MD, USA). The vector containing the Klf2 3′UTR or mutant 3′UTRs and miR-29c-3p sense sequences or a negative control miRNA were co-transfected into HEK293T cells (*n* = 6 per group) and the luciferase activity was assessed 48h later. Gaussia Luciferase activity was measured using the Secrete-Pair™ Dual Luminescence Assay Kit (GeneCopoeia, Rockville, MD, USA) and read on the SpectraMax M5. All measurements were performed in triplicate and normalized to SEAP (secreted Alkaline Phosphatase) activity.

### 4.5. Isolation and Culture of EPCs

Mouse EPCs were isolated using the mouse Lineage Cell Depletion Kit (Miltenyi Biotec Inc, Auburn, CA, USA) and grown in endothelial basal medium-2 (EBM™-2 (Lonza, Tampa, FL, USA)) supplemented with EGM™-2 MV Microvascular Endothelial SingleQuots™ Kit (Lonza) [8]. All in vitro assays conducted in this work were performed with these cells.

### 4.6. MiRNA and shRNA Modulation in EPCs

To study the biological effect of miRNA-mRNA pairs, lentiviral vectors containing pre-miR mimic or anti-miR miRNA inhibitors (System Biosciences Inc (SBI), Palo Alto, CA, USA), mRNAs (GeneCopoeia, Rockville, MD, USA), or shRNAs for specific mRNA or scrambled shRNA (Santa Cruz Biotechnology, Dallas, TX, USA) were transfected into EPCs. Lentiviruses were packaged in 293T cells [38]. The transduction efficiency of EPCs was monitored by the percentage of GFP-positive cells. Stably transduced cells were established in culture under puromycin (5 μg/mL) selection.

### 4.7. Self-Renewal Assay

Self-renewal potential was examined by colony formation assay as previously described [39].

### 4.8. In Vitro Angiogenesis Assay

In vitro angiogenic activity was determined by Matrigel tube formation assay as previously described [40].

### 4.9. Western Blotting

Western blots were performed according to [8]. Primary antibodies for p-ERK1/2 and ERK1/2, were purchased from Cell Signaling. Anti-spred1 was purchased from Abcam, and rabbit polyclonal anti-GAPDH (Sigma-Aldrich, St. Louis, MO, USA) was used as a loading control. For detection, secondary antibodies were horseradish peroxidase-conjugated anti-rabbit (1:4000) or anti-mouse (1:5000) antibodies. Bands were visualized with Super Signal West Femto Maximum Sensitivity Substrate (Thermo Fisher Scientific, Waltham, MA, USA) and quantified with Quantity One System (Bio-Rad Laboratories, Hercules, CA, USA).

### 4.10. Myocardial Ischemia (MI) Model and Cell Therapy

Myocardial ischemia/infarction (MI) has major consequences on the functionality of the heart and can cause permanent and irreversible tissue damage, increasing the risk of developing further cardiovascular pathologies, such as heart failure [23]. Further, MI represents a well-established model to evaluate the efficacy of cellular and other therapeutic modalities designed to treat cardiovascular disease [41]. Male C57BL/6 (10–12 weeks) mice were anesthetized (isoflurane chamber—isoflurane mixed with oxygen 3–5% for anesthesia followed by facemask with isoflurane at 2% mixed with Oxygen), orally intubated, and placed in a supine position. Anesthesia was confirmed by assessing reflexes (pedal, tail), mucous membranes color and observing animal’s breathing before starting the procedure. Animals were monitored during surgery every 10 min for anesthetic state. Left thoracotomy was performed in the fourth intercostal space and the left anterior descending (LAD) branch of the coronary artery was ligated using an 8–0 nylon suture. Coronary artery occlusion was verified by elevation of the ST segment in the ECG. Heart left ventricular function was evaluated echo imaging was implemented by using Visual Sonics, Vevo-770 system (Toronto, ON, Canada). Contractile parameters were calculated automatically from short axis images. Basically, the M-mode tracings were used to measure LV end-diastolic diameter (EDD), end-systolic diameter (ESD), and systolic and diastolic posterior wall thickness, averaging three cardiac cycles. Fractional shortening (FS) was calculated using the following equation: FS% = (LVEDD-LVSDD)/LVEDD × 100. Before ligation, mice received 1 × 10^6^ genetically modified EPCs in 150 μL EBM™-2 through a single tail vein injection. High-definition echo imaging was implemented using a Visual Sonics Vevo-770 system. Contractile parameters were calculated automatically from short axis images. Endpoint measurements at 8 weeks after ligation were compared among different groups.

### 4.11. Histological Examination

After echo imaging, mice were perfused with PBS followed by DiI solution (1,1′-dioctadecyl-3,3,3′,3′-tetramethylindocarbocyanine perchlorate) to label blood vessels. The heart was fixed by perfusion with 4% paraformaldehyde, extracted, and embedded in paraffin. Sections (8 μm thick) were processed for immunostaining. Masson’s Trichrome staining (Sigma) was used to determine collagen content of the infarct regions. For each heart, 8 to 10 sections from apex to base (1–2 mm apart) were analyzed to calculate the fibrotic and nonfibrotic areas, as well as ventricular and septal wall thickness using PathScan Enabler IV Pathology Slide Scanner.

Infarct fraction was determined as [fibrotic area/(fibrotic + nonfibrotic area)] × 100% as previously described [42]. To detect microvascular density in the peri-infarct area, blood vessels with red fluorescence were examined by conventional and confocal fluorescence microscopy.

The number of vessels and the cumulative vessels lengths were measured using ImageJ software versions 1.45 [43].

### 4.12. Statistical Analyses

Students’ *t*-tests were applied to compare the quantitative measurements in different groups. Correlations between more than 2 groups were performed by one-way analysis of variance (ANOVA) followed by multiple comparisons tests. Statistical analysis was performed using SPSS 16.0 computer software (SPSS Inc., Chicago, IL, USA). *p* < 0.05 was considered statistically significant.

## 5. Conclusions

Our findings are consistent with miR-126a playing a key role in EC-mediated vascular repair and angiogenesis. MiR-126a plays a critical role in endothelial cell proliferation, migration, and angiogenesis as well as response to injury [14]. Targeted deletion of miR-126a in mice impairs vascular integrity and reduced angiogenesis following MI [14]. Within developing aortic arch blood vessels of zebrafish, Klf2 upregulated miR-126a expression leading to VEGF-induced angiogenesis through the suppression of Spred-1 expression [11]. Further, miR-126a protects against vascular injury by promoting EPC migration/homing of EPCs to sites of injury in rats [24]. Our study not only provided additional evidence supporting the protective role of miRNA-126a in vascular repair but also identified important upstream regulators of miRNA-126a in EPCs.

Built on miRNA and mRNA profiling studies in young and aged EPCs, we revealed that miR-29c-3p and miR-126a working in tandem to modify VEGF signaling. Several studies suggested that miR-29c plays a multifactorial role in cardiac health. For example, miR-29c directly targeted insulin growth factor 1 (IGF-1) in HUVECs resulting in reduced migration and tube formation [25]. MiR-29c may be a potential biomarker for cardiovascular diseases, including coronary heart disease, heart failure, and chronic obstructive pulmonary disease (COPD) [26,27]. MiR-29c was responsive to hypoxia-inducible factor (HIF)-1. Upregulated miR-29c suppressed Serca2 expression and the uptake of Ca^2+^ to reduce cardiomyocyte contractility [28]. MiR-29c was also shown to be upregulated in response to TGFb and target the histone lysine methyl transferase 5 (KMT5) altering H4K20me3 levels and inducing cardiac aging [29]. Our study is the first to show that miR-29c expression modulates angiogenesis by regulating the expression of a second miRNA, miR-126a, through repressing the transcriptional activator Klf2. Based on the centrality of miR-29c-3p and miR-126a on cardiovascular health, the miR-29c-3p *→* ↓Klf2 *→*↓miR-126a *→* ↑Spred1 *→*↓VEGF signaling pathway may serve as a potent target pathway for the regulation of EPC functionality and cardiac repair.

## Data Availability

The data underlying this article will be shared on reasonable request to the corresponding author.

## Supplementary Materials

The following supporting information can be downloaded at: https://www.mdpi.com/article/10.3390/ijms26094259/s1.

## Abbreviations

AMI	Acute Myocardial Infarction
CFU	Colony-forming unit
COPD	Chronic Obstructive Pulmonary Disease
CVD	Cardiovascular disease
ECG	Electrocardiogram
ECs	Endothelial cells
EPCs	Endothelial Progenitor Cells
Gluc	Gaussia Luciferase
Hmga2	High-mobility group AT-hook 2
Klf2	Kruppel-like Factor 2
KMT5	Histone lysine methyl transferase 5
LAD	Left anterior descending branch of the coronary artery
Lin- BMCs	Lineage negative bone marrow cells
LVEDd	Left Ventricular End-Diastolic Diameter
MEEBO	Mouse Exonic-Evidence Based-Oligonucleotide
MI	Myocardial infarction
Plk2	Polo-like Kinase 2
PVDF	Polyvinylidene fluoride
SCF	Stem cell factor
SEAP	Alkaline Phosphatase
TUNEL	Terminal deoxynucleotidyl transferase dUTP nick end labeling
