# Supplementary material for: MicroRNA-29c-3p and -126a Contribute to the Decreased Angiogenic Potential of Aging Endothelial Progenitor Cells
Source: Int J Mol Sci. 2025 Apr 30;26(9):4259. doi: 10.3390/ijms26094259 (PMC12072698; doi:10.3390/ijms26094259)
Supplement: Supplementary file 1 [file ijms-26-04259-s001.zip › ijms-3571156-supplementary.pdf]

Supplemental Figures

Supplemental Figure S1. miR-29c-3p and miR-126a regulate tube formation. A) Aged EPCs transduced with lentivirus expressing a miR-29c-3p antagonist (miRZip-29c-3p), Klf2, miR-126a, or Spred-1 silencing (Spred-1 shRNA) showed significantly improved vascular tube formation, as measured by vascular tube number, compared to their respective controls. B) Young EPCs transduced with lentivirus encoding miR-29c-3p, silencing of Klf2 (Klf2 shRNA), a miR-126a agonist (miRZip-126a), or overexpression of Spred1 led to reduced capillary tube numbers compared to the appropriate control treated samples (mean ± standard deviation, $**p<0.01$,$***p<0.001$,$****<0.0001$). Graph shows 3 biological replicates, i.e. assessments for 3 different cell dishes, with 3 to 12 random fields. Test used: ANOVA followed by Dunnett's multiple comparisons test. EPCs isolated from male C57BL/6 mice bone marrow were used in these assays.

Supplemental Figure S2. Mice receiving aged cells transfected with miRZip-29c, Klf2, miR-126a, or Spred-1 siRNA had less fibrosis in a LAD model. A) Treatment with aged EPCs transduced with a miR-29c-3p antagonist (miRZip-29c-3p), Klf2 overexpression, miR-126a overexpression, or Spred-1 silencing (Spred-1 shRNA) led to increased Dil staining compared to the empty vector transduced aged EPCs. B) Treatment with young EPCs transduced with miR-29c-3p overexpression, Klf2 silencing (Klf2 shRNA), miR-126a silencing (miRZip-126a), or Spred-1 overexpression led to decreased Dil staining compared to the empty vector transduced young EPCs (mean ± standard deviation, $*p<0.05$). Graph shows 3 biological replicates, i.e. assessments for 3 different cell dishes. Test used: ANOVA followed by Dunnett's multiple

comparisons test. EPCs isolated from male C57BL/6 mice bone marrow were used in these assays. Scale bars: 50µm.

Supplemental Figure S3. Mice receiving aged cells transfected with miRZip-29c, Klf2, miR-126a, or Spred-1 siRNA and reduced apoptosis in a LAD model. A) Treatment with aged EPCs transduced with a miR-29c-3p antagonist (miRZip-29c-3p), Klf2 overexpression, miR-126a overexpression, or Spred-1 silencing (Spred-1 shRNA) led to increased apoptosis compared to the empty vector transduced aged EPCs, as measured by TUNEL staining. B) Treatment with young EPCs transduced with miR-29c-3p overexpression, Klf2 silencing (Klf2 shRNA), miR-126a silencing (miRZip-126a), or Spred-1 overexpression led to decreased apoptosis compared to the empty vector transduced young EPCs (mean ± standard deviation, $*p<0.05$). Graph shows 3 biological replicates, i.e. assessments for 3 different cell dishes. Test used: ANOVA followed by Dunnett's multiple comparisons test. EPCs isolated from male C57BL/6 mice bone marrow were used in these assays. Scale bars: 50µm.

A

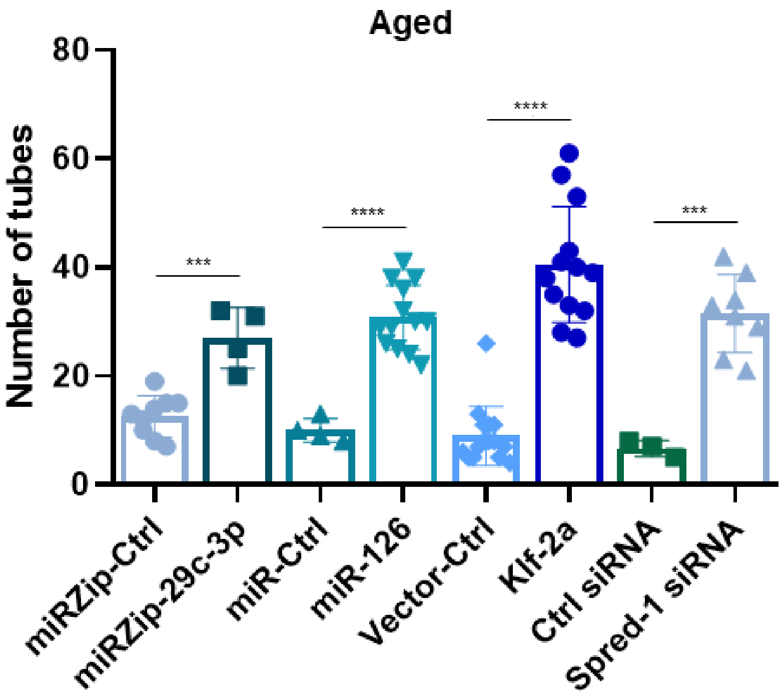
Aged
Number of tubes
80
60
40
20
0
***
****
****
***
miRZip-Ctrl
miRZip-29c-3p
miR-Ctrl
miR-126
Vector-Ctrl
Klf-2a
Ctrl siRNA
Spred-1 siRNA

B

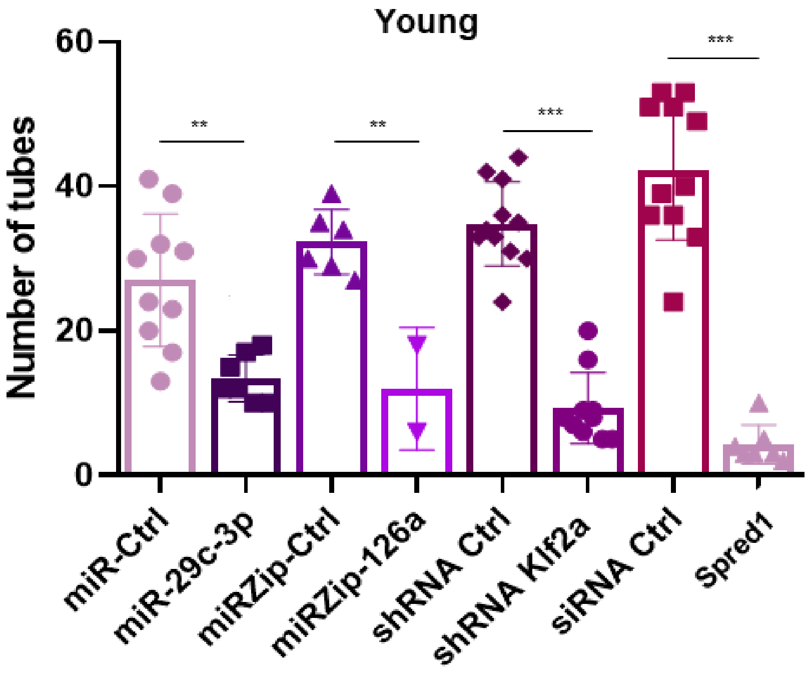
Young
Number of tubes
60
40
20
0
**
**
***
***
miR-Ctrl
miR-29c-3p
miRZip-Ctrl
miRZip-126a
shRNA Ctrl
shRNA Klf2a
siRNA Ctrl
Spred1

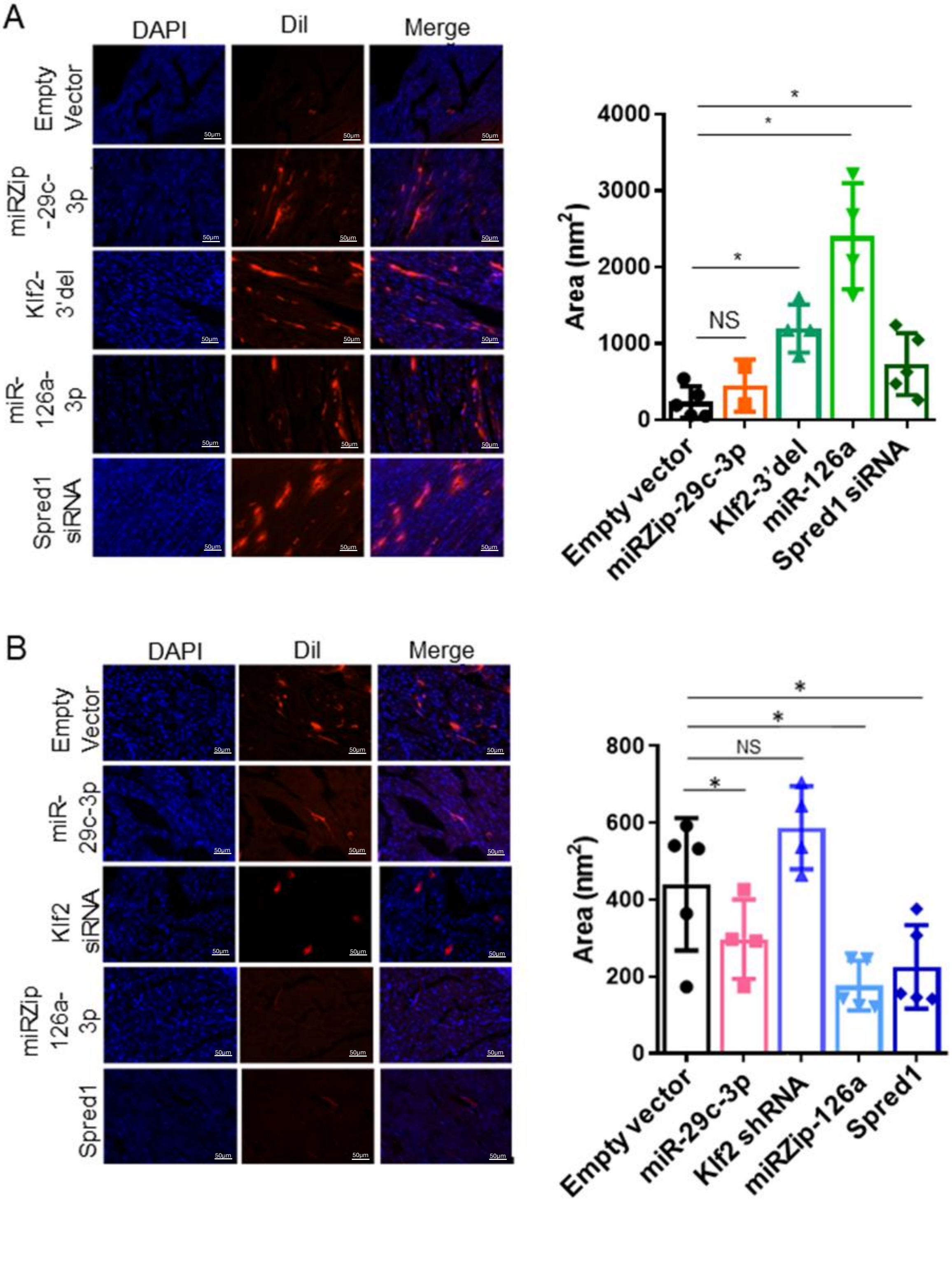
A
DAPI
Dil
Merge
Empty Vector
miRZip -29c-3p
Klf2-3'del
miR-126a-3p
Spred1 siRNA
50μm
Area (nm2)
4000
3000
2000
1000
0
*
NS
Empty vector
miRZip-29c-3p
Klf2-3'del
miR-126a
Spred1 siRNA
B
DAPI
Dil
Merge
Empty Vector
miR-29c-3p
Klf2 siRNA
miRZip 126a-3p
Spred1
50μm
Area (nm2)
800
600
400
200
0
*
NS
Empty vector
miR-29c-3p
Klf2 shRNA
miRZip-126a
Spred1

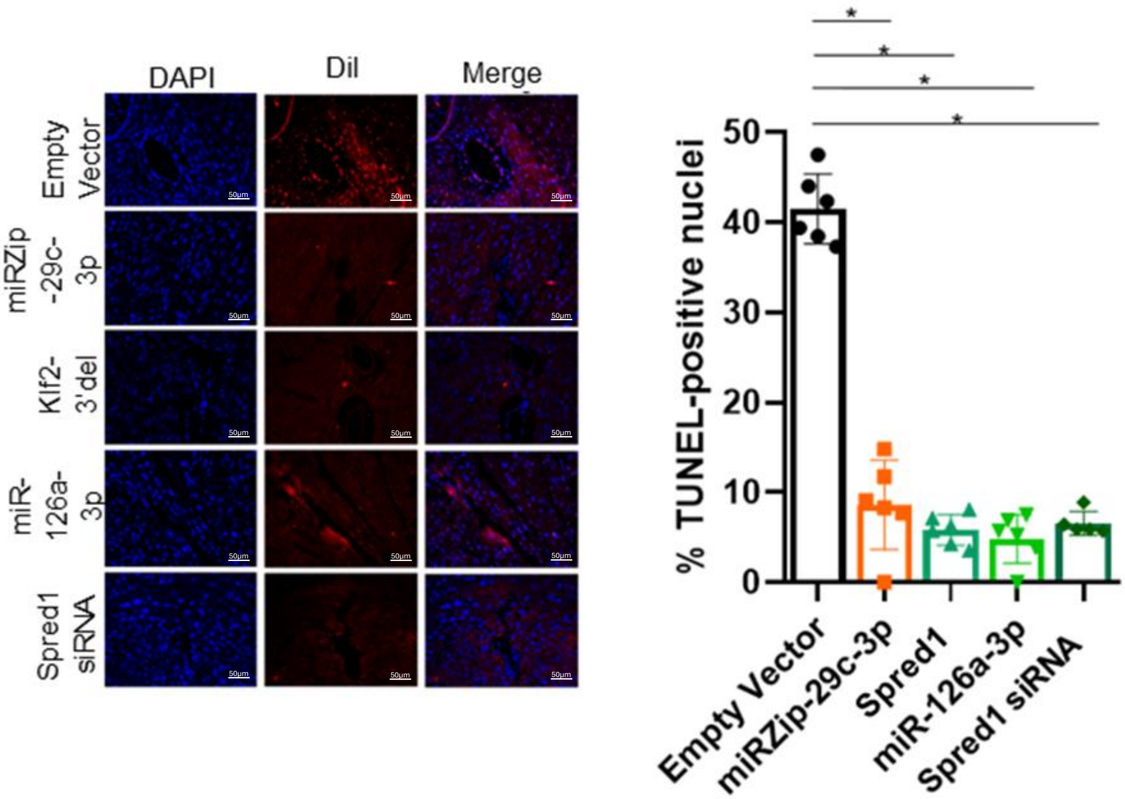
DAPI
Dil
Merge
Empty Vector
miRZip-29c-3p
Klf2-3'del
miR-126a-3p
Spred1 siRNA
50µm
% TUNEL-positive nuclei
0
10
20
30
40
50
*
*
*
*
Empty Vector
miRZip-29c-3p
Spred1
miR-126a-3p
Spred1 siRNA

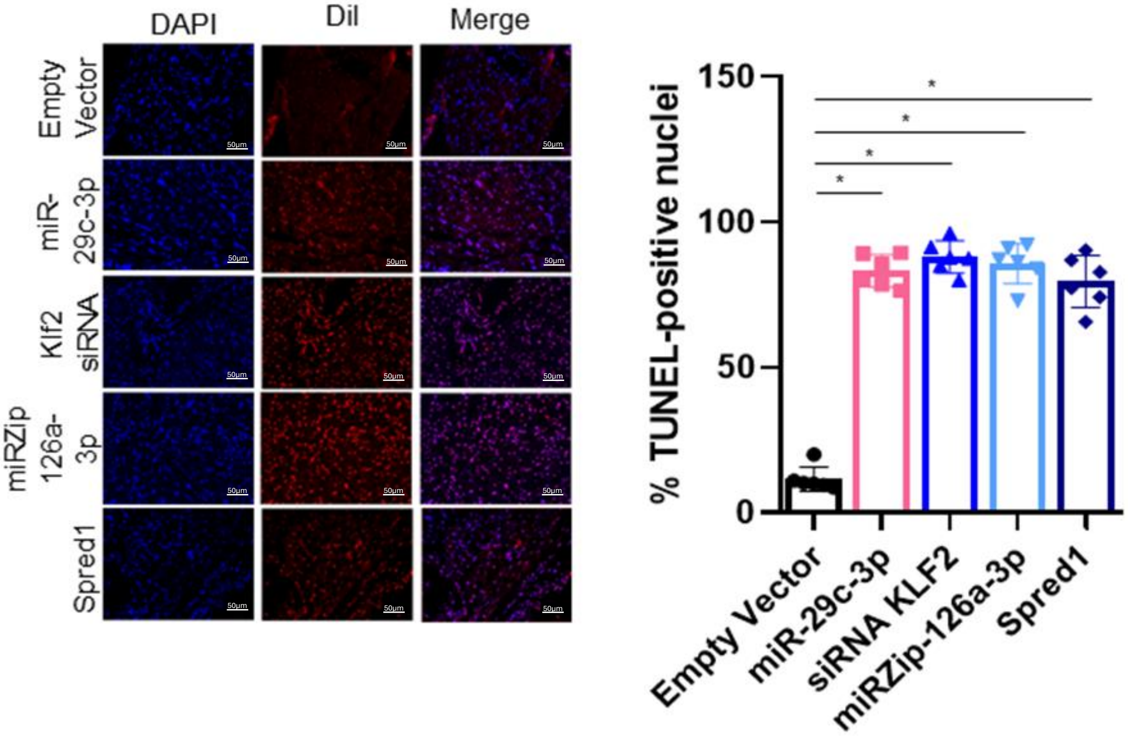
DAPI
Dil
Merge
Empty Vector
miR-29c-3p
Klf2 siRNA
miRZip 126a-3p
Spred1
50µm
% TUNEL-positive nuclei
0
50
100
150
*
*
*
*
Empty Vector
miR-29c-3p
siRNA KLF2
miRZip-126a-3p
Spred1
